# Emerging *Helicobacter pylori* levofloxacin resistance and novel genetic mutation in Nepal

**DOI:** 10.1186/s12866-016-0873-6

**Published:** 2016-11-04

**Authors:** Muhammad Miftahussurur, Pradeep Krishna Shrestha, Phawinee Subsomwong, Rabi Prakash Sharma, Yoshio Yamaoka

**Affiliations:** 1 Department of Environmental and Preventive Medicine, Oita University Faculty of Medicine, 1-1 Idaigaoka, Hasama-machi, Yufu-City, Oita 879-5593 Japan; 2 Gastroenterology Department, Maharajgunj Medical Campus, Tribhuvan University Teaching Hospital, Kathmandu, 44600 Nepal; 3 Department of Gastroenterology and Hepatology, Baylor College of Medicine and Michael DeBakey Veterans Affairs Medical Center, Houston, TX 77030 USA; 4 Gastroentero-Hepatology Division, Department of Internal Medicine, Faculty of Medicine-Institute of Tropical Disease, Universitas Airlangga, Surabaya, 60115 Indonesia

**Keywords:** Nepal, Drug resistance, *Helicobacter pylori*, Genetic mutation

## Abstract

**Background:**

The prevalence of *Helicobacter pylori* antibiotic susceptibility in the Nepalese strains is untracked. We determined the antibiotic susceptibility for *H. pylori* and analyzed the presence of genetic mutations associated with antibiotic resistance in Nepalese strains.

**Results:**

This study included 146 consecutive patients who underwent gastroduodenal endoscopy in Kathmandu, Nepal. Among 42 isolated *H. pylori*, there was no resistance to amoxicillin and tetracycline. In contrast, similar with typical South Asian patterns; metronidazole resistance rate in Nepalese strains were extremely high (88.1 %, 37/42). Clarithromycin resistance rate in Nepalese strains were modestly high (21.4 %, 9/42). Most of metronidazole resistant strains had highly distributed *rdxA* and *frxA* mutations, but were relative coincidence without a synergistic effect to increase the minimum inhibitory concentration (MIC). Among strains with the high MIC, 63.6 % (7/11) were associated with frameshift mutation at position 18 of *frxA* with or without *rdxA* involvement. However, based on next generation sequencing data we found that one strain with the highest MIC value had a novel mutation in the form of amino acid substituted at Ala-212, Gln-382, Ile-485 of *dppA* and Leu-145, Thr-168, Glu-117, Val-121, Arg-221 in *dapF* aside from missense mutations in full-length *rdxA.* Mutations at Asn-87 and/or Asp-91 of the *gyrA* were predominantly in levofloxacin-resistant strains. The *gyrB* mutation had steady relationship with the *gyrA* 87–91 mutations. Although three (44.4 %) and two (22.2 %) of clarithromycin resistant strains had point mutation on A2143G and A2146G, we confirmed the involvement of *rpl22* and *infB* in high MIC strains without an *23SrRNA* mutation.

**Conclusions:**

The rates of resistance to clarithromycin, metronidazole and levofloxacin were high in Nepalese strains, indicating that these antibiotics-based triple therapies are not useful as first-line treatment in Nepal. Bismuth or non-bismuth-based quadruple regimens, furazolidone-based triple therapy or rifabutin-based triple therapy may become alternative strategy in Nepal.

**Electronic supplementary material:**

The online version of this article (doi:10.1186/s12866-016-0873-6) contains supplementary material, which is available to authorized users.

## Background

The achievement of *Helicobacter pylori* against very hostile environment colonized on the stomach of over half of the world's population enact as the most successful human pathogens coexisted nearly sixty thousands years [1]. Although most of individuals exhibit overt disease leading to the hypothesis that the bacterium might be harmless and commensally, chronic infection of *H. pylori* represents a key factor in the etiology of various gastrointestinal diseases including chronic gastritis, peptic ulcer and mucosa-associated lymphoid tissue lymphoma. The outcome of each individual infection is capricious, similar to the rate of progression of the gastric mucosal damage. However, further progression is halted by eradication [2]. A recent meta-analysis supported that *H. pylori* eradication adequately decreases the rate of gastric malignancy, and the magnitude of the protective impact is more noteworthy among individuals with higher baseline gastric cancer risk [3]. Nevertheless, the adequacy of the standard first-line regimen containing a proton pump inhibitor, amoxicillin (AMX) and clarithromycin (CAM) or metronidazole (MNZ) has been seriously challenged and eradication rates below 70 % have been accounted in numerous countries, including South Asia [4, 5].

*H. pylori* antibiotic resistance mechanisms have been recognized in view of the different site-specific mutations that can be distinguished by molecular methods. It is important as a premise for consideration of more rational antibiotic combinations. One mechanism of CAM resistance has been elucidated due to one of five well-known point mutations (A2142G, A2143G, A2142C, A2144T, T2717C and C2694A) in the *23SrRNA* [6, 7]. Our previous report demonstrated higher MICs associated with the synergic effect of mutated sequences in *infB* (*hp1048*), *rpl22* (*hp1314*) and A2143G [8]. Additionally, inactivation mutation including frameshift mutation, insertions and deletions of the *rdxA* (*hp0954*) and *frxA* (*hp0642*) [9]. Novel mutations including *rpsU* (*hp0562*) [10], *dppA* (*hp0298*), *dppB* (*hp0299*), *rps4* (*hp1294*), *ackA (hp0903), rnc (hp0662)* and *dapF (hp0566)* were associated with MNZ resistance [11]. On the other hand, the mechanism of fluoroquinolone resistance in *H. pylori* has been identified to be linked to mutations in the quinolone resistance determining regions of the *gyrA* and *gyrB*, coding of the DNA gyrase [12]. Dual mutations in *gyrA* is accounted for a greater impact, while *gyrB* frequently occurred alongside *gyrA* mutations [13].

Nepal is a small landlocked country in South Asia with a low incidence of gastric cancer (5.3 cases per 100,000 populations per year; GLOBOCAN 2012; http://globocan.iarc.fr). Although it was varied between studies (16.3–70.5 %) [14–19], we confirmed the prevalence of *H. pylori* infection is 38.4 % (56/146) using several diagnostic test that significantly related to source of drinking water [20]. The majority of strains are so-called Western-type-*cagA* in Nepal as similar to typical South Asian patterns [20]. However, the mountainous people of northern Kathmandu are culturally linked to the Buddhists of Tibet, have higher prevalence of *H. pylori* infection and high-risk gastric mucosal atrophy than those Kathmandu people, the capital and the largest urban agglomerate of Nepal [21]. It is suggested lay stress on the need for *H. pylori* eradication in Nepal. Local antibiotic resistances screening are a key to counter primary *H. pylori* treatment failure, thus, reduce possibility spreading of secondary antibiotic resistance [4].

The prevalence of *H. pylori* antibiotic susceptibility in the Nepalese strains is untracked. Table 1 summarized *H. pylori* antibiotics resistance rates in South Asia. Generally, South Asian countries are the high CAM and MNZ resistance prevalence region [5]. Moreover, India and Bangladesh strains demonstrated emerging levofloxacin (LVX) resistance [22, 23], the second-line regimen drug and as a rescue treatment for *H. pylori* eradication*.* In recent years, antibiotic resistance is expanding overall [24, 25], it is critical to look at current drug resistance rates in Nepal. In this study, we aimed to determine the antibiotic susceptibility of *H. pylori* to CAM, MNZ, AMX, tetracycline (TCN), and LVX. Furthermore, we also determined the presence of genetic mutations associated antibiotic resistance in Nepalese strains.

**Table 1:** *H. pylori* antibiotics resistance rates in South Asia

Ref	Country	City	Year	Patients	Methods	CAM	MNZ	LVX	TCN	AMX	Others
[22]	India	Gujarat	2008–2011	80	DDM	58.8 %	83.8 %	72.5 %	53.8 %	72.5 %	Ciprofloxacin (50 %)
[33]	India	Multicentre	–	259	E-test	44.7 %	77.9 %	–	–	32.8 %	–
[53]	India	Kolkata	2000–2001	67	ADM	0.0 %	85.1 %	–	7.5 %	0.0 %	Furazolidone (0.0 %)
[37]	India	North India	–	68	ADM	11.8 %	48.5 %	–	16.2 %	17.6 %	Furazolidone (22.1 %)
[34]	India	Varanasi	2005–2006	63	ADM	4.7 %	100.0		0.0 %	65.1 %	–
[32]	Pakistan	Karachi	2005–2008	178	NM	36.0 %	89.0 %	–	12.0 %	37.0 %	Ofloxacin (18.5 %)
[54]	Pakistan	Karachi	2008–2013	92	E-test	5.4 %	97.8 %	16.2 %	4.3 %	2.2 %	Ofloxacin (30.1 %), Furazolidone (15.2 %)
[55]	Pakistan	Karachi	2007–2009	92	E-test	32.6 %	47.8 %	–	–	2.2 %	–
[56]	Pakistan	Karachi	2009–2010	162	E-test	37.0 %	–	–	–	–	Fluoroquinolone 62.3 %
[35]	Pakistan	Rawalpindi	2011–2012	46	E-test	47.8 %	73.9 %	–	4.4 %	54.3 %	Ciprofloxacin (13.0 %)
[57]	Bangladesh	Dhaka	1999–2001	174	ADM	10.0 %	77.5 %	–	15.0 %	6.6 %	–
[23]	Bangladesh	Dhaka	2014	56	ADM	39.3 %	94.6 %	66.1 %	0.0 %	3.6 %	–

*Abbreviations*: *ADM* Agar Dilution Method, *DDM* Disk diffusion method, *E-test* Epsilometer test, *CAM* clarithromycin, *MNZ* metronidazole, *LVX* levofloxacin, *AMX* amoxicillin, *TCN* tetracycline

## Methods

### Patients and H. pylori

This study included 146 consecutive patients (76 women and 70 men; mean age of 42.2 ± 15.7 years) consecutively from July 2012 to September 2012. The survey was conducted at the endoscopy services section of the Gastroenterology Department, Tribhuvan University Teaching Hospital (TUTH), Kathmandu, Nepal. Peptic ulcer diseases, including gastric and duodenal ulcers, were diagnosed by endoscopic observation, while chronic gastritis was determined by histologic examination. Exclusion criteria included a history of partial gastric resection, eradication therapy for *H. pylori*, and treatment with bismuth-containing compounds, H2-receptor blockers, or proton pump inhibitors (PPI) within four weeks before the study.

For *H. pylori* culture, antral biopsy specimens were homogenized and inoculated onto Mueller Hinton II Agar medium (Becton Dickinson, NJ, USA) supplemented with 7 % horse blood without antibiotics. The plates were incubated for up to 10 days at 37 °C under microaerophilic conditions (10 % O_2_, 5 % CO_2_, and 85 % N_2_). *H. pylori* isolates were identified based on colony morphology; Gram staining results; and positive reactions for oxidase, catalase, and urease. Isolated strains were stored at −80 °C in Brucella Broth (Difco, NJ, USA) containing 10 % dimethyl sulfoxide and 10 % horse serum.

### Antibiotic susceptibility testing

E-test (Biomerieux, Marcy l'´Etoile, France) was used to determine the minimum inhibitory concentration (MIC) of AMX, MNZ, TCN, CAM, and LVX. Mueller-Hinton II Agar medium (Becton Dickinson) supplemented with 10 % defibrinated horse blood was used as culture media. The bacterial suspension, adjusted to be equivalent to a McFarland opacity standard of 3.0, was inoculated onto the plates. After 72 h of incubation, the MIC of each antibiotic was determined. Quality control was performed using *H. pylori* ATCC 43504. The resistance breakpoints were determined as described by the European Committee on Antimicrobial Susceptibility Testing (EUCAST; available in http://www.eucast.org/). Strains were considered to be resistant for MICs >0.125 mg/L for AMX, 0.25 mg/L for CAM, 8 mg/L for MNZ, and 1 mg/L for TCN and LVX.

### Molecular detection on resistant strains

Mutations in *gyrA, gyrB, rdxA, frxA* and *23S rRNA* were assessed on antibiotic-resistant strains by polymerase chain reaction (PCR) based sequencing. *H. pylori* DNA was extracted from *H. pylori* cultured to confluence on MNZ-resistant strains, *gyrA* and *gyrB* for LVX-resistant strains and *23S rRNA* peptidyl transferase for CAM-resistant strains were amplified using the primers on the Additional file 1: Table S1 as described previously [13, 26, 27]. As a control, we sequenced randomly selected 4-sensitive MNZ and LVX strains and 2-sensitive CAM strains. The PCR products were analyzed by gel electrophoresis using 1.5 % agarose gel containing ethidium bromide. The sequences were then generated to the published sequence of the *H. pylori* strain 26695 (GenBank accession number AE000511.1 GI: 6626253) using the MAFFT version 7 (available in http://mafft.cbrc.jp/alignment/server/) and confirmed by visual inspection.

To find other genetic mutations with high MIC values but not involving typical *23S rRNA*, *rdxA* and *frxA* mutations, we also obtained full-length *23S rRNA, infB*, *rpl22* [8], *rdxA, frxA, rpsU* [10], *dppA*, *dppB*, *rps4*, *ackA, rnc* and *dapF* [11] from next-generation sequencing (NGS) data (MiSeq next-generation sequencer; Illumina, Inc., San Diego, CA). MiSeq output was integrated into contig sequences by CLC Genomics Workbench 7.0.4. Genomics Workbench was also used for gene prediction and translation to protein sequences.

### Statistical analysis

Discrete variables were tested using the chi-square test, while continuous variables were tested using the Mann–Whitney *U* and *t*-tests. *P* values < 0.05 were considered statistically significant. The SPSS statistical software package version 18.0 (SPSS, Inc., Chicago, IL) was used for all statistical analyses.

## Results

### Prevalence of antibiotic resistance

**Fig. 1:**
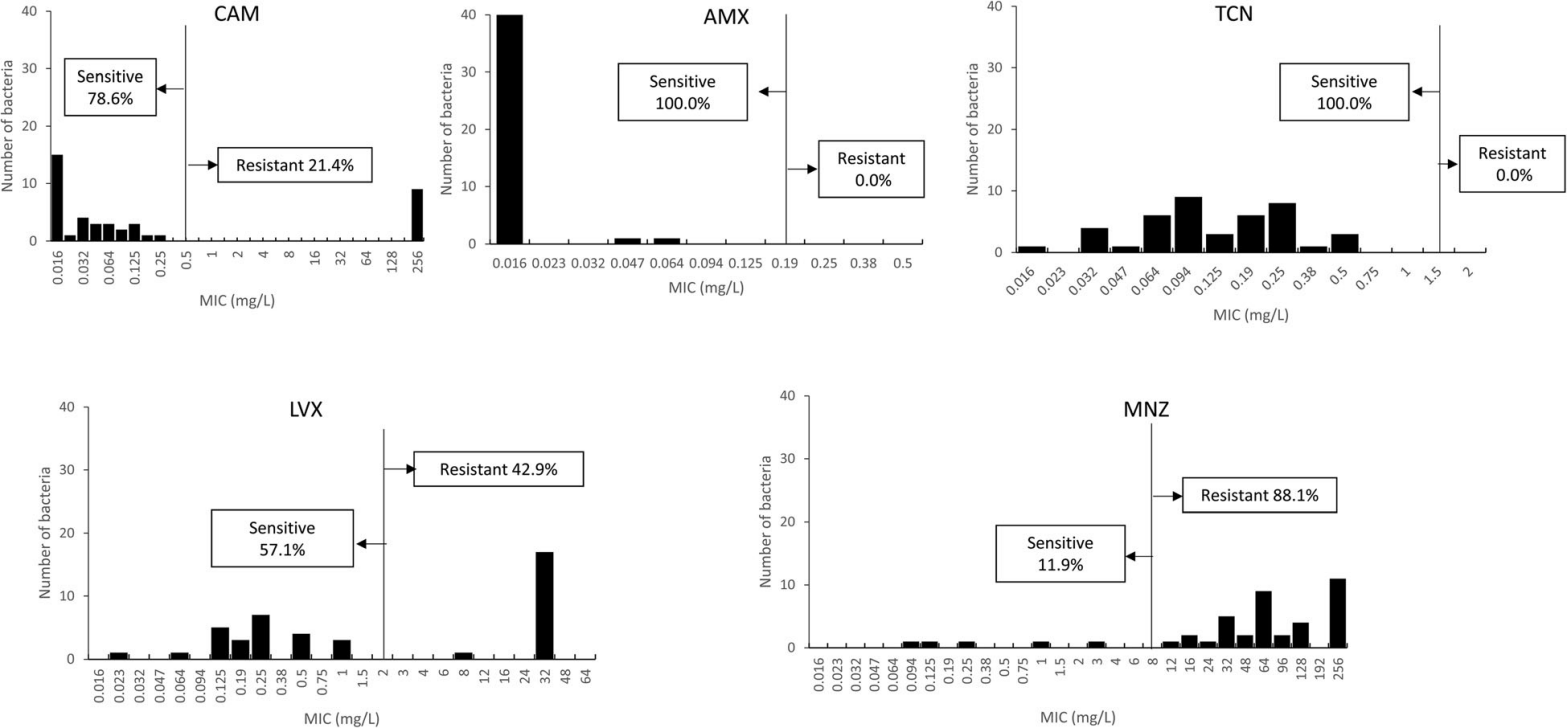
Distribution of antibiotic MIC values. The resistance rates to clarithromycin, metronidazole, levofloxacin were high; in contrast with other South Asian countries, resistance rates to amoxicillin and tetracycline were very low

The prevalence of *H. pylori* infection was 37.7 % (55/146) based on histology confirmed by immunohistochemistry, whereas using culture it was 34.9 % (51/146) [20]. However, 9 isolates did not grow when subcultured onto Mueller Hinton II Agar medium from antibiotic selection plate. Finally, a total of 42 *H. pylori* strains were successfully isolated; consisting 16 male (age range, 17 to 77 years; mean age, 42.3 ± 18.9 years) and 26 female patients (age range, 17 to 69 years; mean age 43.3 ± 14.8 years). The patients consisted of 35 with chronic gastritis, 4 with peptic ulcer diseases and 3 with gastric cancer. Overall, only three strains showed sensitive to all antibiotics (7.14 %). Interestingly, there was no AMX- and TCN-resistant strains and these strains had low MIC predominant (90.5 % for 0.016 mg/L or less for AMX and for 0.25 mg/L or less for TCN, respectively) (Table 2). In contrast, similar with typical South Asian pattern [5]; MNZ resistance rate in Nepalese strains showed an emerging antimicrobial resistance pattern (88.1 %, 37/42) with MIC values 64 mg/L or more (26/37, 70.3 %, Fig. 1). In addition, although CAM resistance rate in Nepalese strains were modestly high (21.4 %, 9/42), we detected a high prevalence of LVX resistance (42.9 %, 18/42) with a high distribution of great MIC values predominant (94.4 % of resistant strains showed 32 mg/L or more). Antibiotic resistance rate did not differ among different age groups, gender and clinical outcomes (*P* >0.05).

**Table 2:** The distribution of antibiotic resistance of *H. pylori* Nepalese isolated strains by sex and age

Antibiotic	All patients	Sex		Age (years)				
		Female	Male	<29	30–39	40–49	50–59	>60
	(*n* = 42)	(*n* = 26)	(*n* = 16)	(*n* = 10)	(*n* = 7)	(*n* = 11)	(*n* = 7)	(*n* = 7)
AMX	0 (0.0)	0 (0.0)	0 (0.0)	0 (0.0)	0 (0.0)	0 (0.0)	0 (0.0)	0 (0.0)
CAM	9 (21.4)	7 (26.9)	2 (12.5)	2 (20.0)	2 (28.6)	2 (18.2)	1 (14.3)	2 (28.6)
MNZ	37 (88.1)	24 (92.3)	13 (81.3)	8 (80.0)	6 (85.7)	9 (81.8)	7 (100.0)	7 (100.0)
TNC	0 (0.0)	0 (0.0)	0 (0.0)	0 (0.0)	0 (0.0)	0 (0.0)	0 (0.0)	0 (0.0)
LVX	18 (42.9)	10 (38.5)	8 (50.0)	6 (60.0)	2 (28.6)	3 (27.3)	2 (28.6)	5 (71.4)

*Abbreviations*: *AMX* amoxicillin, *CAM* clarithromycin, *MNZ* metronidazole, *TCN* tetracycline, *LVX* levofloxacin

Overall, there was no strain resistant to all tested antibiotics. Only five strains were resistant to triple antibiotics; CAM, MNZ, and LVX (Table 3). Among all strains, 28.6 % (12/42) showed dual-drug resistance to MNZ and LVX. Additionally, three strains (7.1 %) were resistant to CAM and MNZ. No differences were observed in clinical outcomes between single-drug and multidrug resistant infections (*P* >0.05).

**Table 3:** The antibiotic resistance patterns of *H. pylori* Nepalese strains

Resistance pattern	N
Double drugs	
MNZ + LVX	12 (28.6)
MNZ + CAM	3 (7.1)
Triple drugs	
CAM + MNZ + LVX	5 (11.9)

*Abbreviations*: *CAM* clarithromycin, *MNZ* metronidazole, *LVX* levofloxacin

### Detection of *H. pylori* genes mutations associated with antimicrobial resistance

The two and three MNZ-resistant strains did not show PCR identifiable specific bands target of *rdxA* and *frxA*, respectively. Therefore, a total 35 *rdxA* and 34 *frxA* of MNZ-resistant strains were analyzed in this study compared to 4-sensitive strains. Both of DNA sequence analysis of *rdxA* and *frxA* from MNZ-sensitive strains revealed intact reading frames (lacking nonsense mutations). Pairwise alignment identified that the MNZ-sensitive strains shared 94.5–97.3 % and 96.5–98.6 % identity with the reference strain, 26695 for *rdxA* and *frxA*, respectively. In contrast, most of the *rdxA* of MNZ-resistant strains contained missense mutations (12/37, 32.4 %) and nonsense mutation resulted premature stop codon (12/37, 32.4 %, Table 4). Moreover, *rdxA* alleles of 7 strains (18.9 %) contained nucleotide deletion and/or insertion that resulted in translational frameshift. The similar pattern with *rdxA* showed in *frxA* of MNZ-resistant that also contained missense mutations, premature stop codon and translational frameshift (11/37, 29.7 %; 4/37, 10.8 % and 17/37, 45.9 %, respectively). The association between these two genes was relative coincidence without a synergistic effect to increase MIC values. Among 7 strains with high MIC values (>256 mg/L or more), 63.6 % strains were associated with frameshift mutation at position 18 of *frxA* (7/11) with or without *rdxA* involvement. Interestingly, there was no mutation on any *rdxA* and *frxA* in one strain with high MIC values (Nepal120).

**Table 4:** MIC of metronidazole resistant strains and the mutation of *rdxA* and *frxA* genes

No	Strains	MIC (mg/L)	*rdxA*	*frxA*
1	2	48	13frameshift	R86^a^
2	4	>256	Q11^a^	18frameshift
3	5	64	N73^a^	R3T, 54frameshift
4	8	64	Q11^a^	Q5^a^
5	14	32	R16H, L62V, K190^a^	P2E, R3P, M66I, A70V
6	15	64	K2N, 4frameshift	I44T, 47frameshift
7	16	48	E107R, 109frameshift	W137^a^
8	18	128	C148Y	undetermined
9	29	>256	R16H, A80T, S108A	18frameshift
10	34	64	undetermined	106frameshift
11	41	16	R16H, R41K, 43frameshift	G76R, A152V
12	49	>256	C140Y	18frameshift
13	52	>256	G189S	undetermined
14	55	24	L62V, S108A, S196N, Q197^a^	P41L, E176K
15	61	12	None	A15V, I144V, M66I
16	64	32	R16H, S108A, R176C, S196N	None
17	70	32	K60^a^	D2E, A85V, K178N
18	74	64	S45G	6frameshift
19	83	>256	M21V, A80T, Q119^a^	A70V
20	84	16	Q50^a^	R58H
21	86	96	Q50^a^	R25T, M66I, A154T
22	89	32	Q65^a^	A115V
23	90	64	45frameshift	18frameshift
24	92	>256	C140Y	18frameshift
25	94	64	Q50^a^	undetermined
26	108	128	R16L	18frameshift
27	110	32	None	P41L
28	113	128	A40T	A16T, I44V, 70frameshift
29	114	>256	Q16^a^	18frameshift
30	116	>256	G163D	18frameshift
31	120^b^	>256	None	None
32	123	>256	Q50^a^	V6^a^
33	124	>256	D23G	18frameshift
34	137	128	M56I, 201frameshift	70frameshift
35	140	96	60frameshift	71frameshift
36	141	64	S43L	72frameshift
37	142	64	undetermined	A15V

Q11^a^ means premature stop codon at Gln11; 13frameshift means frameshift mutation in the amino acid 13; R16H means amino acid substituted at Arg-16; None means no specific mutation; Undetermined is the strains that failed to show identifiable specific bands of *rdxA* or *frxA* target in PCR

^b^High MIC values strain without specific mutation in *rdxA* and *frxA* but contained mutation in *dppA* and *dapF*

Based on the previous report [10, 11], we performed NGS of the Nepal120 strain (average sequencing depth was 249.8× and overall %GC was 39.0). Nonetheless, we could not obtain *ackA* and *rnc* from NGS data. Using strain 26695 and the control MNZ-sensitive strain Nepal145, we could not identify any mutations in full-length *frxA, dppB, rpsU* and *rps4*. In contrast, we revealed missense mutations in the full-length of *rdxA* at Arg-90, His-97, Pro-106 and Val-111. Moreover, we also confirmed involvement of novel mutated sequences in the form of amino acid substituted at Ala-212, Gln-382, Ile-485 of *dppA* and Leu-145, Thr-168, Glu-117, Val-121, Arg-221 in *dapF.*

There was no mutation on both of *gyrA* and *gyrB* subunits among the control four LVX-sensitive strains. Among 18 LVX-resistant strains, 17 had amino acid variants at *gyrA* subunit (Table 5). The major well-known point mutations in the 91- and 87-positions were predominant (15/18, 83.3 %), including 9 of LVX-resistant strains (50.0 %) substituted amino acid at Asp-91, while six strains had amino acid substitution at Asn-87 (33.3 %). Other mutations included substituted amino acid at Ala-88, Ser-63 and Arg-130. On the other hand, only one strain exhibited amino acid substitution at Glu-483 in *gyrB* subunits. However, it is coincidence with *gyrB* without influence to increase of MIC values. There was no correlation between degree of LVX-resistance with the type and number of mutations in both genes.

**Table 5:** MIC of levofloxacin resistant strains and the mutation of *gyrA* and *gyrB* genes

No	Strains	MIC (mg/L)	*gyrA*	*gyrB*
1	2	>32	N87K	None
2	5	>32	D91G	None
3	8	>32	D91N	None
4	16	>32	S63P, D91N	None
5	18	>32	D91N	None
6	29	>32	S63P, N87K, P188S	None
7	38	>32	D99V	None
8	49	>32	N87K, D91N, V172I	None
9	55	>32	N87I	E483K
10	70	>32	None	None
11	86	>32	N87K	None
12	89	>32	D91N, R130K	None
13	90	>32	N87K	None
14	120	8	A88P	None
15	123	>32	D91Y	None
16	140	>32	D91N	None
17	141	>32	D91N	None
18	142	>32	S63P, R130K	None

N87K means amino acid substituted at Asn-87; None means no specific mutation

Based on *23S rRNA* sequenced in the 9 CAM-resistant strains exhibited 3 (44.4 %) and 2 (22.2 %) had point mutation specifically on A2143G and A2146G, respectively. In contrast, we identified minimal nucleotide variation on the CAM-sensitive strains. Interestingly, there was no *23S rRNA* mutation in four strains with high MIC values (>256 mg/L or more). Based on the previous report [8], we also performed next generation sequencing of the Nepal90, Nepal110, Nepal114 and Nepal145 strains (average sequencing depth was 139.5×, 117.3×, 127.5×, 139.4×, respectively and overall %GC was 39.2, 39.0, 38.8, 38.9, respectively). Using strain 26695 and the control CAM-sensitive strain Nepal44, we could not identify any mutations in full-length *23S rRNA*. We confirmed the involvement of novel mutated sequences in C113T and G20A of *rpl22* and some interest mutations of *infB* such as G793A, C2669T, G2043T and C2784A (Table 6).

**Table 6:** MIC of clarithromycin resistant strains and the mutation of *23S rRNA* gene

No	Strains	MIC (mg/L)	*23S rRNA*	*rpl22*	*infB*
1	5	>256	A2143G		
2	29	>256	A2143G		
3	49	>256	A2146G		
4	89	>256	A2143G		
5	90	>256	None	C113T	C193A, T449C, G793A, T870G, C1157T, C1988T, C2669T, A2781G, C2784A
6	92	>256	A2146G		–
7	110	>256	None	None	C133G, G139A, C821T, A2551G, 547del, 571del
8	114	>256	None	G20A	A298G, G448A, G568A, A1108G, G2403T, C2669T
9	145	>256	None	G20A	G8A, A403G, G793A, C810A, C878T, T1171G, G2043T, C2784A, G793A, C812T

A2143G means point mutation at 2143 position; None means no specific mutation

## Discussion

The AMX resistance rates in South Asia is diverse (Table 1), we revealed there was no AMX resistance from Nepalese isolates. Together with CAM or MNZ, AMX is the first-line regimen for treatment of *H. pylori* infection particularly as a secondary antibiotic in the low efficacy of CAM-based triple treatment zone [28–31]. Although in general the AMX resistance is rare, the increasing AMX primary resistance rates have been reported in the neighbor’s country; India and Pakistan [22, 32–35]. AMX is one of the most commonly used antibiotics in recent years in Nepal as similar as ceftriaxone and gentamycin [36]. Additionally we observed no resistance to TCN, in contrast to studies from India and Pakistan [22, 32, 35, 37]. TCN is used as a salvage quadruple therapy [28, 38] and may be a useful alternative first-line regimen in Nepal. A strict regulation for anti-microbial use is necessary to counteract failure of these two essential antibiotics in Nepal.

Importantly, we observed a high prevalence of CAM resistance (21.4 %) in Nepalese strains. It is overabundance of the breaking points required by the Maastricht guidelines on *H. pylori* infection management (>15–20 %) [38, 39], consequently, CAM-based regimen may insufficient as a first line treatment for *H. pylori* eradication in Nepal. A meta-analysis demonstrated that utilization of triple therapy that consist of PPI, AMX, and CAM in cases of CAM resistance diminished the treatment efficacy by 66 % [40]. CAM is not a drug of choice in Nepalese physicians related a high cost [41]. Nonetheless, other macrolides consumption such as erythromycin and azithromycin used for lower respiratory infection in Nepal [41] and become essential risk for cross-resistance to CAM [42]. Additionally, similar with other countries in Asia, there was emerging resistance to MNZ in Nepal. MNZ is a simple medication often utilized to treat different diseases, for example, intestinal parasites and periodontal and gynecologic [43, 44]. In Asia, only Japan, Thailand, and Malaysia have populations with <40 % MNZ resistance [5]. Therefore, regimens including MNZ are not suitable and should not be chosen as first-line treatment in Nepal.

The T2183C and A2223G transformations have been frequently found to be the reason of observed CAM resistance in Asian countries than those in Europe and North America [45]. However, in Nepal we observed the contribution of interest point change on A2143G and A2146G, as previous reports [46, 47]. The A2143G mutation has a much stronger effect than the A2142G and A2142C mutations [46]. Interestingly among several strains with high MIC values (>256 mg/L or more) without *23S rRNA* involvement, we confirmed novel mutated sequences in *rpl22* and *infB* in the different position than previous publication [8]. Suggesting that *rpl22* and *infB* mutations might not only result in synergistic effects, but also could be independent causes of CAM resistance. On the other hand, we recognized diverse mutations involving the *rdxA* and *frxA* in the large part MNZ-resistant-strains; appear differently in relation to against MNZ-sensitive strains. Additionally, several strains with high MIC values were associated with a framing error in position 18 of *frxA* that may become a particular mutation site of Nepalese MNZ-resistant strains. Finally, we introduced the novel mutation in *dppA* and *dapF* in addition to *rdxA* mutations but irrespective of *frxA* and *rpsU* mutations. Unlike *dapF* which is associated with biosynthesis of lysine and peptidoglycan [48], *dppA* has a role in the transportation of dipeptide ATP-binding cassette on a drug efflux pump [11] that eventually lead to MNZ resistance.

Several guidelines proposed that LVX ought to be utilized as a part of rescue treatment based on antibiotic susceptibility testing [28, 38, 49]. However, our findings showed a high prevalence of primary resistance to LVX that may also prompt cross-resistance with other fluoroquinolones. It is become a serious challenge and may reduce the efficacy of treatment with LVX-based regimens in Nepal. In addition, together with MNZ, LVX is the most commonly observed as multidrug resistance in Nepal. Furthermore, 5 strains were identified resistance to triple antibiotics. *H. pylori* strains harboring triple or quadruple resistance can hinder the choice and achievement of eradication regimens.

As similar with previous reports [50–52], point mutations at amino acid 87 (Asn to Lys, Tyr, or Ile) and 91 (Asp to Asn, Gly, or Tyr) were also mainly found for Nepalese strains. Interestingly, different transformations including substituted amino acid at Ser-63 and Arg-130 also associated with high MIC values. A few mutations and the coincidence of Glu-483 substitution in *gyrB* subunits with *gyrA* suggested a minimum influence of the *gyrB* mutations in Nepalese LVX-resistant strains. Finally, mutation analysis at position 18 of *frxA*, Asn-87 and/or Asp-91 of *gyrA*, A2143G and A2146G of *23SrRNA* will be useful as guiding follow-up of eradication after first-line regimens failure in Nepal. Recently, it was created a high accuracy DNA strip genotyping test combining PCR and hybridization that allows the molecular identification of mutations in the *gyrA* and *23SrRNA* within 6 h [47].

The number of samples in this study was relatively low, which certainly suggests the limitations of this study. In addition, we only determined the presence of well-known genetic mutations associated with antibiotic resistance. However, our results could as a susceptibility-guided treatment in Nepal. High prevalence of CAM, MNZ and LVX resistance in Nepal results in prerequisite for utilizing other alternative strategies, for example, bismuth or non-bismuth-based quadruple regimens or rifabutin-based triple therapy is fundamental in Nepal (Table 7) [5]. Additional clinical trials are required to enhance the rate of successful eradication in Nepal.

**Table 7:** Regions with reported resistance and potential rescue regimens for *H. pylori* eradication in Asia [5]

Resistance type	Country	First- and second-line therapy							Rescue therapy	
		CAM-based triple therapy	MNZ-based triple therapy	BIS-based quadruple therapy	non-BIS quadruple `concomitant` therapy	furazolidone-based triple therapy	Sequential therapy	Hybrid therapy	LVX-based triple therapy	RIF-based triple therapy
Low resistance to four antibiotics	Taiwan, Thailand, Malaysia	√	√	√	√	√	√	√	√	√
High CAM resistance (>20 %)	Japan		√	√	√	√	√	√	√	√
High MNZ resistance (>40 %)	China-Hong Kong, Saudi Arabia, Singapore, Bhutan	√		√	√	√	√	√	√	√
High CAM and MNZ resistance	Turkey, Bahrain, Vietnam			√	√			√	√	√
High CAM and LVX resistance	South Korea		√	√	√	√	√	√		√
High CAM, MNZ, and LVX resistance	China-Beijing and Southeast China, Bangladesh, Nepal			√	√	√				√
High CAM, MNZ, and AMX resistance	Indonesia			√		√		√	√	√
High CAM, MNZ, AMX, and LVX (CIP) resistance	Iran, India, Pakistan			√						√

*Abbreviations*: *CAM* clarithromycin, *MNZ* metronidazole, *LVX* levofloxacin, *AMX* amoxicillin, *CIP* ciprofloxacin, *TCN* tetracycline, *RIF* Rifabutin

## Conclusions

We revealed the rates of resistance to CAM, MNZ, and LVX were high in Nepal, which recommends that CAM-, MNZ-, and LVX-based triple therapies are not useful as first-line treatment in Nepal. TCN can be still utilized, albeit local information regarding its successful eradication rate is inadequate. Bismuth or non-bismuth-based quadruple regimens, furazolidone-based triple therapy or rifabutin-based triple therapy may become alternative strategy after first-line regimens failure in Nepal.

## Additional file

Additional file 1: Table S1. The oligonucleotide primers for amplifying r*dxA, frxA, gyrA, gyrB* and *23S rRNA*. (DOCX 14 kb)

## Abbreviations

AMX: Amoxicillin
CAM: Clarithromycin
MIC: Minimum inhibitory concentration
MNZ: Metronidazole
PCR: Polymerase chain reaction
PPI: Proton pump inhibitors
TCN: Tetracycline
TUTH: Tribhuvan University Teaching Hospital.
