# Supplementary material for: Emerging Helicobacter pylori levofloxacin resistance and novel genetic mutation in Nepal
Source: BMC Microbiol. 2016 Nov 4;16:256. doi: 10.1186/s12866-016-0873-6 (PMC5096319; doi:10.1186/s12866-016-0873-6)
Supplement: Additional file 1: Table S1. — The oligonucleotide primers for amplifying rdxA, frxA, gyrA, gyrB and 23S rRNA. (DOCX 14 kb) [file 12866_2016_873_MOESM1_ESM.docx]

Additional file 1: Table S1. The oligonucleotide primers for amplifying r*dxA, frxA, gyrA, gyrB* and *23S rRNA*.

| Gene | Primer | Sequence | PCR product (bp) |
| --- | --- | --- | --- |
| *rdxA* | rdxA-19F | 5’-GCCAGATAGCCAAATGGGGG-3’ | 823 |
|  | rdxA-900R | 5’-GAAACGCTTGAAAACACCCC-3’ |  |
| *frxA* | frxA-F | 5’-TCTCAAGCGGAAAAATCCGG-3’ | 654 |
|  | frxA-R | 5’-GCGAGTTTAGTAGTTTTTAA-3’ |  |
| *gyrA* | gyrA-F | 5’-AGCTTATTCCATGAGCGTGA-3’ | 582 |
|  | gyrA-R | 5’-TCAGGCCCTTTGACAAATTC-3’ |  |
| *gyrB* | gyrB-F | 5’-CCCTAACGAAGCCAAAATCA-3’ | 465 |
|  | gyrB-R | 5’-GGGCGCAAATAACGATAGAA-3’ |  |
| *23SrRNA* | HP23-F | 5’-CCACAGCGATGTGGTCTCAG-3’ | 425 |
|  | HP23-R | 5’-CTCCATAAGAGCCAAAGCCC-3’ |  |
